# CARE Dose 4D combined with sinogram‐affirmed iterative reconstruction improved the image quality and reduced the radiation dose in low dose CT of the small intestine

**DOI:** 10.1002/acm2.12502

**Published:** 2018-12-03

**Authors:** Lin Wang, Shenchu Gong, Jushun Yang, Jie Zhou, Jing Xiao, Jin‐hua Gu, Hong Yang, Jianfeng Zhu, Bosheng He

**Affiliations:** ^1^ Department of Radiology The Second Affiliated Hospital of Nantong University Jiangsu China; ^2^ Department of Epidemiology and Medical Statistics School of Public Health Nantong University Nantong Jiangsu China; ^3^ Department of Pathophysiology Nantong University Medical School Nantong Jiangsu China; ^4^ Clinical Medicine Research Center the Second Affiliated Hospital of Nantong University Nantong Jiangsu China

**Keywords:** CARE Dose 4D, image quality, radiation dose, small intestinal diseases, SAFIRE

## Abstract

**Objective:**

Multislice computed tomography (MSCT) has been used for diagnosis of small intestinal diseases. However, the radiation dose is a big problem. This study was to investigate whether CARE Dose 4D combined with sinogram‐affirmed iterative reconstruction (SAFIRE) can provide better image quality at a lower dose for imaging small intestinal diseases compared to MSCT.

**Methods:**

The noise reduction ability of SAFIRE was assessed by scanning the plain water mold using SOMATOM Definition Flash double‐source spiral CT. CT images at each stage of radiography for 239 patients were obtained. The patients were divided into groups A and B were based on different tube voltage and current or the image recombination methods. The images were restructured using with filtered back projection (FBP) and SAFIRE (S1–S5). The contrast noise ratio (CNR), CT Dose index (CTDI), subjective scoring, and objective scoring were compared to obtain the best image and reformation parameters at different stages of CT.

**Results:**

Twenty‐six restructuring patterns of tube voltage and current were obtained by FBP and SAFIRE. The average radiation dose using CARE Dose 4D combined with SAFIRE (S4–S5) reduced approximately 74.85% compared to conditions where the tube voltage of 100 kV and tube current of 131 mAs for patients with MSCT small intestinal CT enterography at plain CT scan, arterial stage, small intestine, and portal venous phase. The objective and subjective scoring were all significantly different among groups A and B at each stage.

**Conclusions:**

Combination of CARE Dose 4D and SAFIRE is shown to decrease the radiation dose while maintaining image quality.

## 1 INTRODUCTION

The small intestine is the main organ of nutrition and water absorption, and is the longest organ in the digestive tract of the human body.^1^, ^2^ Small intestinal disease is common in the clinic and mainly include small bowel obstruction, ischemia, neoplasm, and inflammatory bowel disease.^3^ Early diagnosis of small intestinal disease is challenging as the organ is intertwined and has a small diameter.^4^, ^5^

The common diagnostic methods such as double‐balloon enteroscopy (DBE) and capsule endoscopy (CE) have a lot of limitations such as complicated operation and high cost, and CT provides a new frame of reference for the diagnosis of small intestinal diseases. With application such as multi slice computed tomography (MSCT), DBE, and CE which has greatly improved the diagnosis and treatment of small intestine diseases.^6^, ^7^, ^8^ MSCT is more precise for assessing the blood supply of mesenteric vessels and walls than CE and DBE, and it is very important for diagnostic localization, qualitative analyses, and etiological diagnosis of intestinal obstruction as well as the detection of external intestinal lesions. MSCT has the advantage of being simpler, more accurate, painless, high economic utility ratio, faster imaging velocity with fewer contraindications than the other methods. However, the problem is that radiation dose for CT is high relative to other imaging modalities. The occurrence rate of a malignant tumor will increase 50 thousand every 100 thousand when the dose of x ray adds 1 mSv according to the International Commission on Radiological Protection.^9^ Eillstein et al.^10^ reported that the risk of cancer relative to ionizing radiation caused by a CT scan check raised from 0.4% to 2.0% in USA at 2007. The risk of suffering cancer reached to 0.6% among the people over age 75 in England caused by diagnostic x ray.^11^ It was proven that the dose of CT was proportional to the square of the tube voltage and the tube current which manifested that the radiation dose could be reduced by reducing the tube current.^12^

Radiation dose of CT accounted for about 50% of the medical radiation dose during the past 20 years.^13^ Reducing the radiation dose of CT while ensuring that the image quality is maintained in agreement with the As Low As Reasonably Achievable (ALARA‐principle) has always been a challenge for medical physicists and radiologists.^14^, ^15^ Iterative reconstruction which can effectively reduce the image noise relative to the traditional filtered back projection (FBP), is widely applied in the technique of CT reconstruction in recent years. Sinogram‐affirmed iterative reconstruction (SAFIRE) is the second generation iterative algorithm based on raw data domain and could eliminate image sharpness and pixel noise to improve image quality and further reduce radiation dose. Studies have shown that iterative reconstruction technique could significantly reduce radiation dose. For example, Christe et al.^16^ considered that the iterative reconstruction technique could reduce 27–70% of radiation dose in CT Scan Chest. In the study of Kalra et al.^17^ SAFIRE was considered to reduce radiation dose with fully diagnostic value in abdominal CT images. The combination of low tube current and iterative reconstruction had also been reported to be effective in radiation dose reduction.^18^, ^19^, ^20^ Gandhi et al.^21^ reported that half‐dose of CT enterography with FBP and SAFIRE was more accurate in the diagnosis of advanced inflammatory ileal disease. However, the image quality and inferior visibility of small anatomic structures are still unsatisfactory. CARE Dose 4D is a four‐dimensional automatic real‐time dose adjustment technology which is put forward by Siemens. The technology can determine the size of patients according to the positioning image, and automatically calculate the required tube current value and CT Dose index (CTDI) at different tube voltages, which will obtain the constant diagnostic image quality of all parts of the body with the lowest radiation Dose. Furthermore, CARE Dose 4D was successful to optimize radiation dose in previous studies.^22^, ^23^ We planned to demonstrate CARE Dose 4D in the application of imaging small intestinal diseases.

In this study, we assessed the effect of SAFIRE on noise reduction with the ordinary water film and selected the adequate tube voltage and current. We also reported on the effects of the combination of CARE Dose 4D (Siemens Healthcare) and SAFIRE on the image quality and radiation dose reduction for small intestinal CT.

## 2 METHODS AND MATERIALS

### 2.A Patients

We collected data from 239 patients with small intestine CT angiography who were admitted to the first people's hospital of Nantong during December 2015 and March 2017. There were 116 males and 123 females with an average age of 56.12 yr. The patients were divided into control group (*n* = 17, with standard dose MSCT) and experimental group (*n* = 222, with low dose MSCT). All patients had signed the consent forms with protocols that were approved by the first people's hospital of Nantong.

### 2.B METHODS

#### 2.B.1 The noise reduction effect of SAFIRE

Siemens second generation dual‐source CT (SOMATOM Definition Flash, Siemens, Healthcare, Ellaingen, Germany) was taken to conduct the CT scan for the random regular water mold under different tube voltage and tube current. The benchmark scanning conditions included 120 kV tube voltage, 200 mAs tube current, 128 × 0.6 mm collimation, and a pitch 0.6. The position was fixed according to the location marker of the water phantom and the scanned area through the motif space. Then the tube voltage of 100, 80, and 70 kV were selected in turn to regulate the tube current so that the CTDI_vol_ increments would decrease by 10% for each measurement. The image was reconstructed with filtered back projection (FBP) and SAFIRE (the intensity of iterative was S1–S5) with a seam thickness of 1 mm and interlamellar spacing of 0.7 mm. The convolution kernels were B31f and I30f. The image was transferred to Syngo via the postprocessing workstation. Thereafter, the axial image of the water phantom with a seam thickness of 3 mm and interlamellar spacing of 3 mm was reconstructed under different conditions. Finally, the limitation of the image noise reduced by SAFIRE was measured and assessed according to the signal noise ratio (SNR) of the water model image, and the contrast noise ratio (CNR) of the image of each image. Fifty square centimeters of region of interest (ROI) was selected. The average CT number and standard deviation within the ROI were measured by one physician twice. The formulas were as follows:

$$\text{SNR}={\text{CT}}_{\mathrm{water}\mathrm{phantom}}/{\text{SD}}_{\mathrm{water}\mathrm{phantom}}$$

$$\text{CNR}=\left({\text{CT}}_{\mathrm{water}\mathrm{phantom}}-{\text{CT}}_{\mathrm{air}}\right)/{\text{SD}}_{\mathrm{air}},$$

wherein, CT is the CT value, and SD is the standard deviation.

#### 2.B.2 Small intestine CT angiography

##### Small intestine filling method

Each patient took 1200 ml 2.5% isotonic mannitol orally. Patients with poor tolerance and significant obstruction took iso‐osmolar contrast medium orally with low intensity and high frequency. All patients with intestinal obstruction were treated with gastrointestinal decompression after examination.

##### Small intestine MSCT angiography

Nine groups (A1–A9) were divided according to the different tube voltages and current, while another 6 groups (B0–B5) were grouped via the image recombination methods. Then patients in the control group were firstly conducted with plain CT scan with 120 V tube voltage, 200 mAs tube current, 32 × 1.2 mm collimation and a pitch of 0.6 (group: A1). Then the patients received a three‐phase enhanced scanning after a high‐pressure injection with 350 mg/ml iopromide at a speed of 3.5 ml/s.^24^, ^25^ Image reconstruction was carried out using FBP with a seam thickness of 1 mm, interlamellar spacing of 0.7 mm and a smooth convolution kernel B31f. At last, the data were transferred to Sygo and processed. Meanwhile, the patients in the experimental group were treated with low dose MSCT who were scanned by a Siemens second generation dual‐source CT based on CARE Dose 4D technique in each stage with the CTDI_vol_ decrease by 10% for each measurement (group: A2–A9,). The images were reconstructed via FBP and SAFIRE with a smooth convolution kernel I30f (group B0: FBP; group B1–B5: SAFIRE). The details of the group are in Table 1.

**Table 1:** The groups divided via different scan conditions and reconfiguration method

Group A	Tube voltage (kV)	Tube current (mAs)	*n*	Group B	Reconfiguration method
A1	120	200	17	B0	FBP
A2	100	295	30	B1	SAFIRE, S1
A3	100	262	30	B2	SAFIRE, S2
A4	100	230	30	B3	SAFIRE, S3
A5	100	196	30	B4	SAFIRE, S4
A6	100	164	30	B5	SAFIRE, S5
A7	100	131	30		
A8	100	98	30		
A9	80	207	12		
Total			239		

##### Measurement parameters and methods

**Figure 1:**
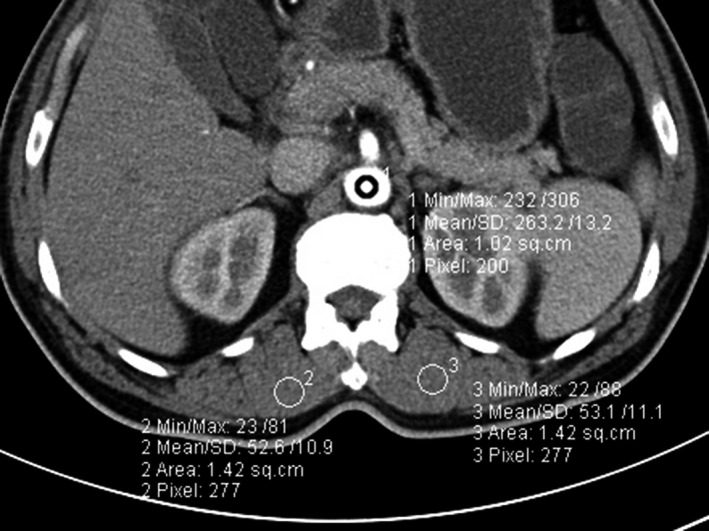
The contrast noise ratio (CNR) of the image at each stage via multislice CT enterography. CNR = (CT _blood vessels_ − CT_muscle_)/SD_muscle_.

**Figure 2:**
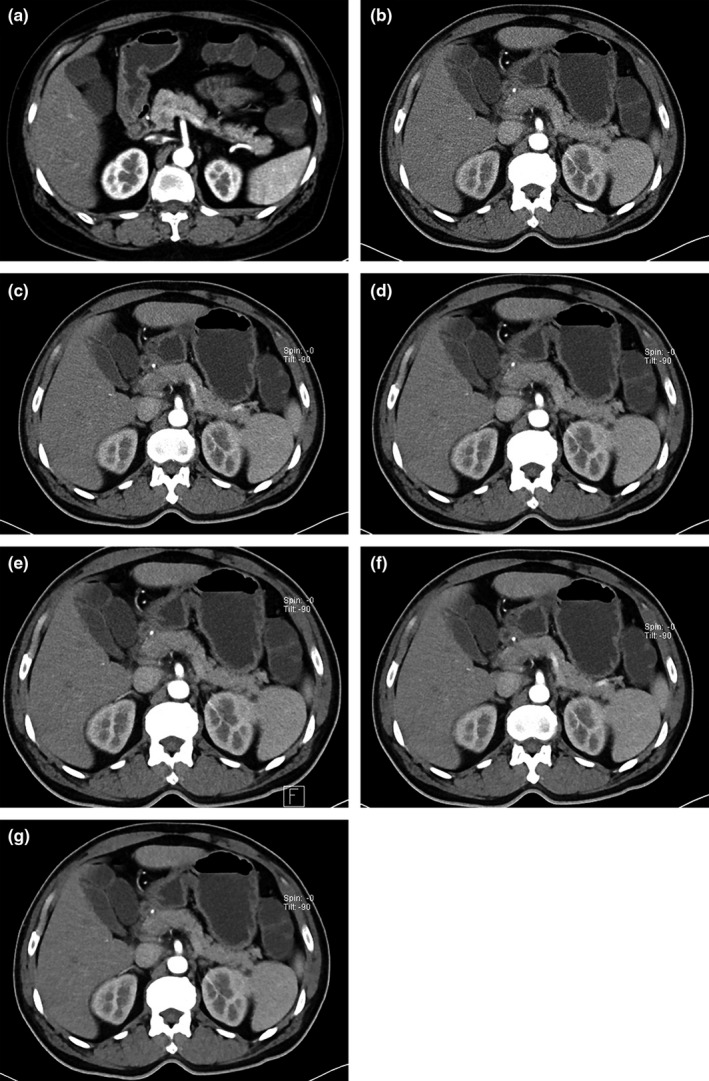
The reconstructed images which was made by FBP under the tube voltage of 120 kV and tube current of 200 mAs (patient: male, 46 yr, BMI = 21.2). (a) The reconstructed images which was made by FBP under the tube voltage of 100 kV and tube current of 131 mAs (patient: male, 46 yr, BMI = 21.2). (b–g). The reconstructed images which was made by SAFIR (S1–S5) under the tube voltage of 100 kV and tube current of 131 mAs (patient: male, 46 yr, BMI = 21.2). BMI, body mass index.

**Figure 3:**
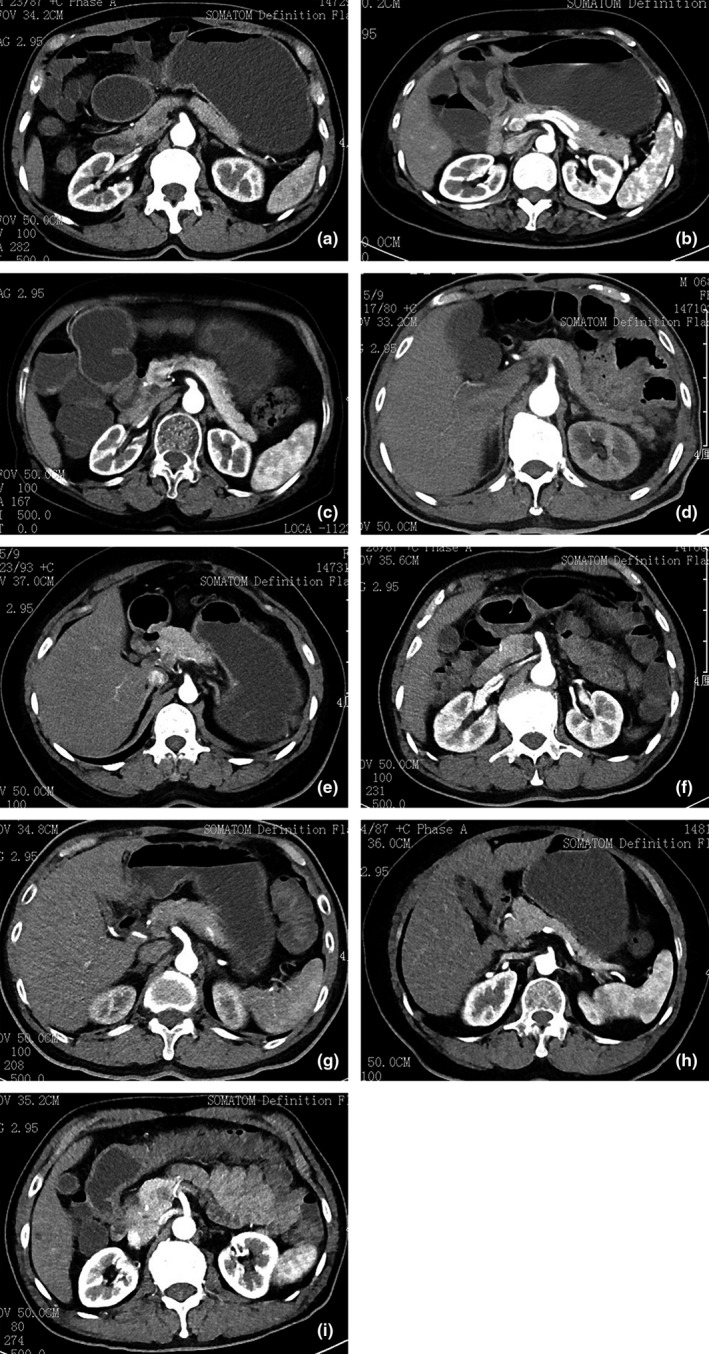
The MSCT radiography of small intestine under different tube voltage and current. (a). The images was reconstructed using FBP under the tube voltage of 120 kV and tube current of 200 mAs at arterial phase (patient A: male, 54 yr, BMI = 21.6). (b)The images was reconstructed by SAFIRE 4 and collected by CARE Dose 4D under the tube voltage of 100 kV and tube current of 295 mAs at arterial phase (patient B: male, 56 yr BMI = 21.3). (c) The images was reconstructed by SAFIRE 4 and collected by CARE Dose 4D under the tube voltage of 100 kV and tube current of 262 mAs at arterial phase (patient C: male, 54 yr, BMI = 21.4). (d) The images was reconstructed by SAFIRE 4 and collected by CARE Dose 4D under the tube voltage of 100 kV and tube current of 230 mAs at arterial phase (patient D: male, 53 yr, BMI = 21.3). (e) The images was reconstructed by SAFIRE 4 and collected by CARE Dose 4D under the tube voltage of 100 kV and tube current of 196 mAs at arterial phase (patient E: male, 55 yr, BMI = 21.7). (f) The images was reconstructed by SAFIRE 4 and collected by CARE Dose 4D under the tube voltage of 100 kV and tube current of 164 mAs at arterial phase (patient F: male, 52 yr, BMI = 21.5). (g) The images was reconstructed by SAFIRE 4 and collected by CARE Dose 4D under the tube voltage of 100 kV and tube current of 131 mAs at arterial phase (patient G: male, 56 yr, BMI = 21.2). (h) The images was reconstructed by SAFIRE 4 and collected by CARE Dose 4D under the tube voltage of 100 kV and tube current of 98 mAs at arterial phase (patient H: male, 53 yr, BMI = 21.3). (i) The images was reconstructed by SAFIRE 4 and collected by CARE Dose 4D under the tube voltage of 80 kV and tube current of 207 mAs at arterial phase (patient I: male, 55 yr, BMI = 21.4). BMI, body mass index

The scan parameters were the same as those of the control group except the tube voltage and tube current. The multiplanar reconstruction (MPR), maximum intensity projection (MIP) and volume rendered technique (VRT) at the axial and coronary position of the small intestinal CT at each stage in two groups were conducted by the postprocessing workstation vascular software. The central level on superior mesenteric artery ostium was chosen as the object of interest and the CNR was calculated as follows: CNR = (CT_blood vessel_ − CT _muscle_)/SD_muscle_ (Fig. 1). According to the indices outlined in the American Association of Physicists in Medicine (AAPM) Report 204,^26^ the conversion factor (*f*) of the CT dose index volume was obtained. The effective tube current (emAs) and the CT dose index volume (CTDI_vol_) were obtained and CTDI_vol_ was taken as the evaluation index of the radiation dose. The SSDE was assessed with the formula (SSDE = CTDI_vol _× *f*) from the AAPM Report 204. The CNR to CTDI_vol_ ratio (CCR) was calculated (CCR = CNR/CTDI_vol_). Combing with the MPR, MIP, and VRT, the visual scores (VS), including the presentation of mesenteric vessels, intestinal wall, and the main substance of the lesion in the scanning area were assessed for the CT image of the small intestine at each stage. According to the European CT image quality guide,^27^ the image noise and quality assessment were conducted based on the visual perception or particle character of the noise and the ability to show the details of the anatomy (Figs. 2 and 3). The visual scores and objective evaluation criteria were measured and analyzed by two radiologists who had 5 years of experience in abdominal imaging diagnosis. The subjective criteria is shown in Table 2.

**Table 2:** The subjective evaluation indexes of multislice CT enterography

Scoring	Mesenteric vessel		Intestinal wall	Substantial organ lesions	Comment
	Number	Shape			
1	Only show level 1 branches	Morphological ambiguity cannot be diagnosed	Only the intestinal wall is full satisfactory (long intestinal tube diameter ≥ 1 cm); The scoring criteria are consistent with the criteria of mesenteric vascular morphology	Nearly can't show	The highest score for each item is scored as the single item; The sum of the scores is the final score of subjective evaluation
2	2 Show level 2 branches	Morphological is not clear, details show bad		It can be displayed, but the edge is not clear	
3	Show level 3 branches	Most of the blood vessel shows clear and minority does not show and cannot be evaluated		Clearly show and sharp‐edged	
4	Show level 4 branches	Shape and detail display are clear and can be evaluated, but not ideal			
5		The vascular morphological details are clear and can be evaluated accurately			

### 2.C Statistical analysis

All data were analyzed by Microsoft Excel 2016 and SPSS 22.0 software (SPSS Inc, Chicago, IL). The Interclass correlation coefficient (ICC) was used to measure the consistency of the results between two observers (ICC > 0.80: well, 0.61–0.80: medium, 0.60 to 0.41: ordinary, ICC < 0.40: poor). The differences in BMI among each group were analyzed by one‐way ANOVA. Two‐way Anova analysis was implemented to analyze the diversity of CNR, CCR, and CTDI_vol_ of _the_ small intestine at each stage under different conditions. The differences between two parameters were compared with a least significant difference. The VS was compared by the rank transformation test; *P *≤* *0.05 was considered to be significant.

## 3 RESULTS

### 3.A SAFIRE–improved image quality with dose reduction

The result by the FBP method of under 120 kV and 200 mAs showed that the CTDI_vol_ was 13.46 mGy and the image noise was 4.00 HU. The SNR and CNR were 0.18 and 459.69, respectively. Taking this result as the standard, we compared the image noise, SNR, and CNR under different scan conditions which were obtained by FBP and SAFIRE (the iterative intensity was between S1 and S5). The noise of water model images were obtained by different scanning conditions and SAFIRE reconstruction conditions should be lower than that of the control group, and SNR and CNR should be higher than that of the control group. The combination of the qualified tube voltage, tube current, and the SAFIRE iteration strength was recorded (Table 3).

**Table 3:** The restructuring way of tube voltage and tube current which were satisfied the conditions

No	Tube voltage (kV)	Tube current (mAs)	Recombination	Noise (Hu)	SNR	CNR	CTDI_vol_ (mGy, %)
1^a^	120	200	FBP	4.00	0.18	459.69	13.46, 100
2	100	295	S1	3.80	0.29	467.54	12.08, 90
3	100	295	S2	3.30	0.33	513.66	12.08, 90
4	100	295	S3	2.80	0.41	619.53	12.08, 90
5	100	295	S4	2.30	0.50	684.33	12.08, 90
6	100	295	S5	2.10	0.24	871.78	12.08, 90
7	100	262	S2	3.50	0.37	512.30	10.74, 80
8	100	262	S3	3.00	0.43	610.29	10.74, 80
9	100	262	S4	2.50	0.52	683.03	10.74, 80
10	100	262	S5	2.00	0.65	853.54	10.74, 80
11	100	230	S2	3.75	0.32	490.31	9.41, 70
12	100	230	S3	3.20	0.41	566.74	9.41, 70
13	100	230	S4	2.70	0.48	644.40	9.41, 70
14	100	230	S5	2.10	0.62	791.25	9.41, 70
15	100	196	S3	3.40	0.38	540.02	8.07, 60
16	100	196	S4	2.80	0.46	605.48	8.07, 60
17	100	196	S5	2.20	0.59	761.09	8.07, 60
18	100	164	S3	3.70	0.38	493.20	6.73, 50
19	100	164	S4	3.10	0.45	574.84	6.73, 50
20	100	164	S5	2.50	0.56	700.86	6.73, 50
21	100	131	S4	3.50	0.43	525.59	5.39, 40
22	100	131	S5	2.70	0.52	654.92	5.39, 40
23	100	98	S5	3.15	0.48	562.58	4.02, 30
24	100	65	S5	3.90	0.38	467.21	2.68 20
25	80	275	S4	3.55	0.20	509.54	5.32, 40
26	80	275	S5	2.80	0.25	624.92	5.32, 40
27	80	207	S5	3.30	0.27	551.64	4.05, 30

a Represented that the image quality and radiation dose under the standard abdominal imaging conditions and this was taken as reference standard. S1–S5 represented the recombination iteration strength of SAFIRE; CTDI: actual measure value,the percentage of measurement in standard radiation dose.

### 3.B The image quality of small intestinal CT radiography and radiation dose at each stage

The consistency of the measurement result and subjective scoring correlated well where the ICC ranged from 0.868 to 0.913. The result of observer A was randomly chosen for subsequent statistical analysis. One‐way Anova demonstrated that there was no statistical difference for BMI of patients between two groups (*P *=* *0.337). Two‐way Anova analysis for two methods of grouping was conducted. The image quality and radiation dose at each stage were shown in Tables 4, 5, 6, 7.

**Table 4:** CT image quality of small intestine and radiation dose — the summary of plain CT scan

A	Tube voltage (kV)	Tube current (mAs)	B	Objective scoring				Subjective scoring		
				CTDI_vol_(mGy)	SSDE (mGy)	CNR	CCR	VS _blood vessel_	VS _intestinal wall_	VS _lesion_
1	120	200	0	13.46 ± 0.00	4.72 ± 1.06	2.73 ± 2.15	0.20 ± 0.16	5.00 ± 0.00	5.00 ± 0.00	5.00 ± 0.00
2	100	295	0	8.22 ± 2.13	11.35 ± 2.88	0.75 ± 0.64	0.10 ± 0.09	3.77 ± 0.43	4.73 ± 0.45	3.00 ± 0.00
2	100	295	1	8.22 ± 2.13	11.35 ± 2.88	0.89 ± 0.59	0.11 ± 0.08	3.77 ± 0.43	4.87 ± 0.35	3.00 ± 0.00
2	100	295	2	8.22 ± 2.13	11.35 ± 2.88	0.96 ± 0.53	0.11 ± 0.04	3.77 ± 0.43	5.00 ± 0.00	3.00 ± 0.00
2	100	295	3	8.22 ± 2.13	11.35 ± 2.88	1.29 ± 1.33	0.15 ± 0.15	3.90 ± 0.31	5.00 ± 0.00	3.00 ± 0.00
2	100	295	4	8.22 ± 2.13	11.35 ± 2.88	1.24 ± 1.20	0.18 ± 0.19	3.77 ± 0.43	5.00 ± 0.00	3.00 ± 0.00
2	100	295	5	8.22 ± 2.13	11.35 ± 2.88	1.08 ± 0.87	0.12 ± 0.08	3.77 ± 0.43	5.00 ± 0.00	3.00 ± 0.00
3	100	262	0	7.82 ± 1.66	11.36 ± 2.73	1.12 ± 1.39	0.14 ± 0.17	4.00 ± 0.00	4.67 ± 0.55	2.90 ± 0.31
3	100	262	1	7.82 ± 1.66	10.97 ± 2.58	1.27 ± 1.22	0.16 ± 0.14	4.00 ± 0.00	4.67 ± 0.55	2.90 ± 0.31
3	100	262	2	7.82 ± 1.66	10.97 ± 2.58	1.46 ± 1.55	0.18 ± 0.19	4.00 ± 0.00	4.67 ± 0.55	2.90 ± 0.31
3	100	262	3	7.82 ± 1.66	10.97 ± 2.58	1.64 ± 1.45	0.20 ± 0.17	4.00 ± 0.00	4.73 ± 0.52	2.93 ± 0.25
3	100	262	4	7.82 ± 1.66	10.97 ± 2.58	1.74 ± 1.78	0.22 ± 0.21	4.00 ± 0.00	4.50 ± 0.68	2.90 ± 0.31
3	100	262	5	7.82 ± 1.66	10.97 ± 2.58	2.06 ± 2.04	0.25 ± 0.24	4.00 ± 0.00	4.50 ± 0.68	2.80 ± 0.41
4	100	230	0	6.38 ± 1.05	9.12 ± 1.61	1.19 ± 0.35	0.19 ± 0.06	4.00 ± 0.00	5.00 ± 0.00	3.00 ± 0.00
4	100	230	1	6.38 ± 1.05	9.12 ± 1.61	1.33 ± 0.48	0.22 ± 0.10	4.00 ± 0.00	5.00 ± 0.00	3.00 ± 0.00
4	100	230	2	6.38 ± 1.05	9.12 ± 1.61	1.45 ± 0.36	0.24 ± 0.09	4.00 ± 0.00	5.00 ± 0.00	3.00 ± 0.00
4	100	230	3	6.38 ± 1.05	9.12 ± 1.61	1.49 ± 0.52	0.25 ± 0.11	4.00 ± 0.00	4.73 ± 0.69	3.00 ± 0.00
4	100	230	4	6.38 ± 1.05	9.12 ± 1.61	1.67 ± 0.90	0.28 ± 0.17	4.00 ± 0.00	5.00 ± 0.00	3.00 ± 0.00
4	100	230	5	6.38 ± 1.05	9.12 ± 1.61	1.75 ± 1.24	0.41 ± 0.24	4.00 ± 0.00	5.00 ± 0.00	3.00 ± 0.00
5	100	196	0	6.39 ± 1.36	8.95 ± 2.18	1.11 ± 1.09	0.18 ± 0.20	4.00 ± 0.00	4.70 ± 0.47	3.00 ± 0.00
5	100	196	1	6.39 ± 1.36	8.95 ± 2.18	1.41 ± 1.17	0.23 ± 0.21	4.00 ± 0.00	4.90 ± 0.31	3.00 ± 0.00
5	100	196	2	6.39 ± 1.36	8.95 ± 2.18	1.49 ± 1.20	0.25 ± 0.25	4.00 ± 0.00	4.90 ± 0.31	3.00 ± 0.00
5	100	196	3	6.39 ± 1.36	8.95 ± 2.18	1.32 ± 1.47	0.22 ± 0.26	4.00 ± 0.00	4.90 ± 0.31	3.00 ± 0.00
5	100	196	4	6.39 ± 1.36	8.95 ± 2.18	2.00 ± 1.69	0.33 ± 0.32	4.00 ± 0.00	4.90 ± 0.31	3.00 ± 0.00
5	100	196	5	6.39 ± 1.36	8.95 ± 2.18	2.53 ± 2.42	0.43 ± 0.48	4.00 ± 0.00	4.90 ± 0.31	3.00 ± 0.00
6	100	164	0	4.27 ± 0.74	6.07 ± 1.16	0.84 ± 0.76	0.20 ± 0.19	4.00 ± 0.00	4.87 ± 0.35	3.00 ± 0.00
6	100	164	1	4.27 ± 0.74	6.07 ± 1.16	0.96 ± 0.79	0.23 ± 0.21	4.00 ± 0.00	5.00 ± 0.00	3.00 ± 0.00
6	100	164	2	4.27 ± 0.74	6.07 ± 1.16	1.08 ± 0.96	0.25 ± 0.23	4.00 ± 0.00	5.00 ± 0.00	3.00 ± 0.00
6	100	164	3	4.27 ± 0.74	6.07 ± 1.16	1.19 ± 0.93	0.29 ± 0.24	4.00 ± 0.00	5.00 ± 0.00	3.00 ± 0.00
6	100	164	4	4.27 ± 0.74	6.07 ± 1.16	1.45 ± 0.99	0.35 ± 0.25	4.00 ± 0.00	5.00 ± 0.00	3.00 ± 0.00
6	100	164	5	4.27 ± 0.74	6.07 ± 1.16	1.56 ± 1.50	0.38 ± 0.38	4.00 ± 0.00	5.00 ± 0.00	3.00 ± 0.00
7	100	131	0	3.29 ± 0.48	4.53 ± 0.75	0.91 ± 0.67	0.29 ± 0.22	4.00 ± 0.00	4.57 ± 0.50	3.00 ± 0.00
7	100	131	1	3.29 ± 0.48	4.53 ± 0.75	0.90 ± 0.65	0.28 ± 0.21	4.00 ± 0.00	4.57 ± 0.50	3.00 ± 0.00
7	100	131	2	3.29 ± 0.48	4.53 ± 0.75	0.86 ± 0.73	0.27 ± 0.23	4.00 ± 0.00	4.67 ± 0.48	3.00 ± 0.00
7	100	131	3	3.29 ± 0.48	4.53 ± 0.75	1.05 ± 0.61	0.32 ± 0.29	4.00 ± 0.00	4.77 ± 0.43	3.00 ± 0.00
7	100	131	4	3.29 ± 0.48	4.53 ± 0.75	1.19 ± 0.86	0.37 ± 0.27	4.00 ± 0.00	4.67 ± 0.48	3.00 ± 0.00
7	100	131	5	3.29 ± 0.48	4.53 ± 0.75	1.55 ± 1.06	0.48 ± 0.32	4.00 ± 0.00	4.57 ± 0.50	3.00 ± 0.00
8	100	98	0	3.04 ± 0.67	4.16 ± 0.94	0.80 ± 0.47	0.27 ± 0.16	3.67 ± 0.48	4.63 ± 0.49	2.87 ± 0.35
8	100	98	1	3.04 ± 0.67	4.16 ± 0.94	0.82 ± 0.59	0.28 ± 0.21	3.87 ± 0.35	4.77 ± 0.43	2.87 ± 0.35
8	100	98	2	3.04 ± 0.67	4.16 ± 0.94	0.92 ± 0.76	0.32 ± 0.28	3.80 ± 0.41	4.63 ± 0.49	2.87 ± 0.35
8	100	98	3	3.04 ± 0.67	4.16 ± 0.94	1.19 ± 0.92	0.42 ± 0.33	3.80 ± 0.41	4.83 ± 0.38	2.87 ± 0.35
8	100	98	4	3.04 ± 0.67	4.16 ± 0.94	1.17 ± 0.74	0.41 ± 0.27	3.80 ± 0.41	4.77 ± 0.43	2.93 ± 0.25
8	100	98	5	3.04 ± 0.67	4.16 ± 0.94	1.71 ± 1.44	0.60 ± 0.32	3.73 ± 0.45	4.77 ± 0.43	2.93 ± 0.25
9	80	207	0	3.25 ± 0.58	4.04 ± 0.71	0.72 ± 0.43	0.22 ± 0.13	3.00 ± 0.00	4.00 ± 0.00	2.00 ± 0.00
9	80	207	1	3.25 ± 0.58	4.04 ± 0.71	0.83 ± 0.76	0.26 ± 0.24	3.00 ± 0.00	4.00 ± 0.00	2.00 ± 0.00
9	80	207	2	3.25 ± 0.58	4.04 ± 0.71	0.76 ± 0.52	0.23 ± 0.15	3.00 ± 0.00	4.00 ± 0.00	2.00 ± 0.00
9	80	207	3	3.25 ± 0.58	4.04 ± 0.71	0.86 ± 0.81	0.26 ± 0.24	3.00 ± 0.00	4.00 ± 0.00	2.00 ± 0.00
9	80	207	4	3.25 ± 0.58	4.04 ± 0.71	1.00 ± 0.75	0.31 ± 0.22	3.00 ± 0.00	4.00 ± 0.00	2.00 ± 0.00
9	80	207	5	3.25 ± 0.58	4.04 ± 0.71	1.32 ± 1.15	0.41 ± 0.35	3.00 ± 0.00	4.00 ± 0.00	2.00 ± 0.00
Total			*F*	18.135	14.425	318.762	219.652	138.335	46.682	224.241
			*P*	0.000	0.000	0.000	0.000	0.000	0.000	0.000
A			*F*	17.607	13.941	512.766	352.211	223.204	74.467	359.466
			*P*	0.000	0.000	0.000	0.000	0.000	0.000	0.000
B			*F*	18.733	17.648	0.000	12.551	0.748	3.237	0.652
			*P*	0.000	0.000	1.000	0.000	0.587	0.007	0.660

**Table 5:** CT image quality of small intestine and radiation dose — the summary of arterial phase

A	Tube voltage (kV)		Tube current (mAs)	B	Objective scoring				Subjective scoring		
					CTDI_vol_ (mGy)	SSDE (mGy)	CNR	CCR	Blood vessel	Intestinal wall	Lesions
1	120		200	0	13.46 ± 0.00	4.63 ± 0.85	47.08 ± 48.92	3.50 ± 3.63	5.00 ± 0.00	5.00 ± 0.00	5.00 ± 0.00
2	100		295	0	7.74 ± 1.44	15.63 ± 4.25	34.15 ± 7.01	4.54 ± 1.21	3.66 ± 0.48	4.86 ± 0.35	3.00 ± 0.00
2	100		295	1	7.61 ± 1.47	10.76 ± 2.31	34.19 ± 6.78	4.68 ± 1.41	3.74 ± 0.44	4.87 ± 0.34	3.00 ± 0.00
2	100		295	2	7.67 ± 1.46	10.64 ± 2.23	34.26 ± 6.73	4.68 ± 1.53	3.77 ± 0.43	5.00 ± 0.00	3.00 ± 0.00
2	100		295	3	7.67 ± 1.46	10.64 ± 2.23	42.68 ± 11.62	5.83 ± 2.06	3.77 ± 0.43	5.00 ± 0.00	3.00 ± 0.00
2	100		295	4	7.67 ± 1.46	10.64 ± 2.23	44.52 ± 10.06	5.93 ± 1.37	3.77 ± 0.43	4.87 ± 0.35	3.00 ± 0.00
2	100		295	5	7.67 ± 1.46	10.64 ± 2.23	45.15 ± 9.11	6.20 ± 3.15	3.77 ± 0.43	5.00 ± 0.00	3.00 ± 0.00
3	100		262	0	7.62 ± 1.59	10.62 ± 2.15	24.34 ± 7.86	3.42 ± 1.61	4.00 ± 0.00	4.53 ± 0.68	3.00 ± 0.00
3	100		262	1	7.62 ± 1.59	10.52 ± 1.90	26.13 ± 9.72	3.67 ± 1.85	4.00 ± 0.00	4.50 ± 0.68	3.00 ± 0.00
3	100		262	2	7.62 ± 1.59	10.57 ± 1.92	28.90 ± 7.44	4.02 ± 1.54	4.00 ± 0.00	4.70 ± 0.65	3.00 ± 0.00
3	100		262	3	7.62 ± 1.59	10.57 ± 1.92	31.32 ± 12.47	4.41 ± 2.25	4.00 ± 0.00	4.70 ± 0.65	3.00 ± 0.00
3	100		262	4	7.62 ± 1.59	10.57 ± 1.92	41.20 ± 11.59	5.70 ± 2.18	4.00 ± 0.00	4.53 ± 0.68	3.00 ± 0.00
3	100		262	5	7.62 ± 1.59	10.57 ± 1.92	45.00 ± 16.44	6.37 ± 3.15	4.00 ± 0.00	4.50 ± 0.68	3.00 ± 0.00
4	100		230	0	6.44 ± 2.52	10.15 ± 3.33	23.43 ± 10.11	4.16 ± 2.45	4.00 ± 0.00	5.00 ± 0.00	3.00 ± 0.00
4	100		230	1	6.44 ± 2.52	9.25 ± 3.67	25.20 ± 10.37	4.50 ± 2.60	4.00 ± 0.00	5.00 ± 0.00	3.00 ± 0.00
4	100		230	2	6.44 ± 2.52	9.21 ± 3.69	27.83 ± 12.60	4.65 ± 2.11	4.00 ± 0.00	5.00 ± 0.00	3.00 ± 0.00
4	100		230	3	6.44 ± 2.52	9.21 ± 3.69	32.54 ± 15.83	5.37 ± 2.68	4.00 ± 0.00	5.00 ± 0.00	3.00 ± 0.00
4	100		230	4	6.44 ± 2.52	9.21 ± 3.69	34.98 ± 18.73	5.84 ± 3.42	4.00 ± 0.00	4.87 ± 0.35	3.00 ± 0.00
4	100		230	5	6.44 ± 2.52	9.21 ± 3.69	35.77 ± 21.11	6.55 ± 4.78	4.00 ± 0.00	5.00 ± 0.00	3.00 ± 0.00
5	100		196	0	6.30 ± 1.25	8.90 ± 2.50	24.74 ± 6.33	4.09 ± 1.41	4.00 ± 0.00	4.77 ± 0.43	3.00 ± 0.00
5	100		196	1	6.30 ± 1.25	8.79 ± 2.01	28.11 ± 7.22	4.66 ± 1.63	4.00 ± 0.00	4.83 ± 0.38	3.00 ± 0.00
5	100		196	2	6.30 ± 1.25	8.81 ± 2.02	30.95 ± 8.40	5.10 ± 1.80	4.00 ± 0.00	4.83 ± 0.38	3.00 ± 0.00
5	100		196	3	6.30 ± 1.25	8.81 ± 2.02	38.64 ± 9.74	6.40 ± 2.19	4.00 ± 0.00	4.83 ± 0.38	3.00 ± 0.00
5	100		196	4	6.30 ± 1.25	8.81 ± 2.02	43.25 ± 10.67	7.13 ± 2.19	4.00 ± 0.00	4.83 ± 0.38	3.00 ± 0.00
5	100		196	5	6.30 ± 1.25	8.81 ± 2.02	47.48 ± 21.71	7.90 ± 4.33	4.00 ± 0.00	4.90 ± 0.31	3.00 ± 0.00
6	100		164	0	3.88 ± 0.73	7.28 ± 2.34	25.50 ± 12.74	6.60 ± 3.34	4.00 ± 0.00	4.80 ± 0.41	3.00 ± 0.00
6	100		164	1	3.88 ± 0.73	5.56 ± 1.02	27.11 ± 13.18	7.03 ± 3.45	4.00 ± 0.00	4.93 ± 0.25	3.00 ± 0.00
6	100		164	2	3.88 ± 0.73	5.50 ± 0.98	32.50 ± 15.23	8.39 ± 4.03	4.00 ± 0.00	4.93 ± 0.25	3.00 ± 0.00
6	100		164	3	3.88 ± 0.73	5.50 ± 0.98	61.72 ± 15.41	8.96 ± 4.16	4.00 ± 0.00	4.90 ± 0.31	3.00 ± 0.00
6	100		164	4	3.88 ± 0.73	5.50 ± 0.98	38.24 ± 19.16	9.79 ± 4.66	4.00 ± 0.00	4.93 ± 0.25	3.00 ± 0.00
6	100		164	5	3.88 ± 0.73	5.50 ± 0.98	47.20 ± 25.65	12.03 ± 6.25	4.00 ± 0.00	4.93 ± 0.25	3.00 ± 0.00
7	100		131	0	3.64 ± 1.40	5.37 ± 1.52	25.73 ± 7.69	7.64 ± 2.86	4.00 ± 0.00	4.63 ± 0.49	3.00 ± 0.00
7	100		131	1	3.64 ± 1.40	5.06 ± 2.10	27.46 ± 7.87	8.18 ± 2.96	4.00 ± 0.00	4.73 ± 0.45	3.00 ± 0.00
7	100		131	2	3.64 ± 1.40	5.06 ± 2.12	29.11 ± 7.90	8.80 ± 2.87	4.00 ± 0.00	4.73 ± 0.45	3.00 ± 0.00
7	100		131	3	3.64 ± 1.40	5.06 ± 2.12	33.66 ± 9.41	9.99 ± 3.39	4.00 ± 0.00	4.73 ± 0.45	3.00 ± 0.00
7	100		131	4	3.64 ± 1.40	5.06 ± 2.12	40.71 ± 11.23	12.17 ± 4.51	4.00 ± 0.00	4.83 ± 0.35	3.00 ± 0.00
7	100		131	5	3.64 ± 1.40	5.06 ± 2.12	49.34 ± 15.78	14.75 ± 5.86	4.00 ± 0.00	4.93 ± 0.25	3.00 ± 0.00
8	100		98	0	2.58 ± 0.47	4.38 ± 1.69	22.35 ± 6.68	8.88 ± 2.73	3.73 ± 0.48	4.57 ± 1.03	2.73 ± 0.69
8	100		98	1	2.58 ± 0.47	3.53 ± 0.73	22.90 ± 7.7	9.20 ± 3.60	3.73 ± 0.48	4.77 ± 0.43	2.73 ± 0.69
8	100		98	2	2.58 ± 0.47	3.53 ± 0.75	26.80 ± 9.36	10.65 ± 3.79	3.73 ± 0.48	4.77 ± 0.43	2.73 ± 0.69
8	100		98	3	2.58 ± 0.47	3.53 ± 0.75	28.50 ± 9.06	11.25 ± 3.57	3.73 ± 0.48	4.77 ± 0.43	2.73 ± 0.69
8	100		98	4	2.58 ± 0.47	3.53 ± 0.75	29.96 ± 11.46	11.91 ± 4.60	3.73 ± 0.48	4.77 ± 0.43	2.73 ± 0.69
8	100		98	5	2.58 ± 0.47	3.53 ± 0.75	33.78 ± 11.01	13.43 ± 4.40	3.73 ± 0.48	4.83 ± 0.38	2.73 ± 0.69
9	80		207	0	3.25 ± 0.58	3.52 ± 0.74	25.92 ± 6.21	8.22 ± 2.34	3.00 ± 0.00	4.00 ± 0.00	2.00 ± 0.00
9	80		207	1	3.22 ± 0.55	3.57 ± 0,81	26.38 ± 6.00	8.41 ± 2.42	3.00 ± 0.00	4.00 ± 0.00	2.00 ± 0.00
9	80		207	2	3.22 ± 0.55	4.54 ± 0.98	24.02 ± 7.03	7.64 ± 2.53	3.00 ± 0.00	4.00 ± 0.00	2.00 ± 0.00
9	80		207	3	3.22 ± 0.55	4.46 ± 0.84	29.18 ± 7.82	9.20 ± 2.75	3.00 ± 0.00	4.00 ± 0.00	2.00 ± 0.00
9	80		207	4	3.22 ± 0.55	4.46 ± 0.84	33.29 ± 5.40	10.61 ± 2.46	3.00 ± 0.00	4.00 ± 0.00	2.00 ± 0.00
9	80		207	5	3.22 ± 0.55	4.46 ± 0.84	37.05 ± 7.15	11.81 ± 3.03	3.00 ± 0.00	4.00 ± 0.00	2.00 ± 0.00
Total		*F*			21.102	9.622	230.332	165.905	137.784	39.851	370.084
		*P*			0.000	0.000	0.000	0.000	0.000	0.000	0.000
A		*F*			25.543	5.647	370.206	266.035	222.207	63.112	594.055
		*P*			0.000	0.000	0.000	0.000	0.000	0.000	0.000
B		*F*			13.092	17.291	0.003	9.842	0.148	3.481	0.000
		*P*			0.000	0.000	1.000	0.000	0.981	0.004	1.000

**Table 6:** CT image quality of small intestine and radiation dose — the summary of small intestine period

A	Tube voltage (kV)		Tube current (mAs)	B	Objective scoring				Subjective scoring		
					CTDI_vol_ (mGy)	SSDE(mGy)	CNR	CCR	Blood vessel	Intestinal wall	Lesions
1	120		200	0	13.46 ± 0.00	4.60 ± 0.82	7.82 ± 2.94	0.58 ± 0.22	5.00 ± 0.00	5.00 ± 0.00	5.00 ± 0.00
2	100		295	0	7.66 ± 1.46	15.58 ± 4.19	11.04 ± 3.07	1.50 ± 0.50	3.77 ± 0.43	4.87 ± 0.35	3.00 ± 0.00
2	100		295	1	7.66 ± 1.46	10.63 ± 2.27	11.32 ± 1.41	1.53 ± 0.38	3.77 ± 0.43	5.00 ± 0.00	3.00 ± 0.00
2	100		295	2	7.66 ± 1.46	10.62 ± 2.23	12.29 ± 3.42	1.69 ± 0.65	3.77 ± 0.43	5.00 ± 0.00	3.00 ± 0.00
2	100		295	3	7.66 ± 1.46	10.62 ± 2.23	17.41 ± 7.34	2.38 ± 1.16	3.77 ± 0.43	5.00 ± 0.00	3.00 ± 0.00
2	100		295	4	7.66 ± 1.46	10.62 ± 2.23	16.57 ± 3.71	2.25 ± 0.70	3.77 ± 0.43	5.00 ± 0.00	3.00 ± 0.00
2	100		295	5	7.66 ± 1.46	10.62 ± 2.23	21.24 ± 10.97	3.03 ± 2.08	3.77 ± 0.43	5.00 ± 0.00	3.00 ± 0.00
3	100		262	0	9.22 ± 2.53	10.47 ± 2.14	7.73 ± 3.26	0.91 ± 0.41	4.00 ± 0.00	4.57 ± 0.68	3.00 ± 0.00
3	100		262	1	9.22 ± 2.53	12.89 ± 3.95	8.72 ± 3.08	1.02 ± 0.43	4.00 ± 0.00	4.70 ± 0.47	3.00 ± 0.00
3	100		262	2	9.22 ± 2.53	12.95 ± 3.95	10.32 ± 3.19	1.20 ± 0.50	4.00 ± 0.00	4.60 ± 0.50	3.00 ± 0.00
3	100		262	3	9.22 ± 2.53	12.95 ± 3.95	11.74 ± 4.51	1.36 ± 0.55	4.00 ± 0.00	4.60 ± 0.50	3.00 ± 0.00
3	100		262	4	9.22 ± 2.53	12.95 ± 3.95	14.71 ± 4.77	1.69 ± 0.61	4.00 ± 0.00	4.70 ± 0.47	3.00 ± 0.00
3	100		262	5	9.22 ± 2.53	12.95 ± 3.95	14.41 ± 5.85	1.65 ± 0.70	4.00 ± 0.00	4.70 ± 0.47	3.00 ± 0.00
4	100		230	0	8.41 ± 2.61	13.52 ± 4.12	6.89 ± 2.26	0.97 ± 0.64	4.00 ± 0.00	4.87 ± 0.35	3.00 ± 0.00
4	100		230	1	8.41 ± 2.61	12.08 ± 3.93	6.77 ± 1.45	0.95 ± 0.56	4.00 ± 0.00	5.00 ± 0.00	3.00 ± 0.00
4	100		230	2	8.41 ± 2.61	12.03 ± 3.92	7.36 ± 2.00	1.06 ± 0.67	4.00 ± 0.00	5.00 ± 0.00	3.00 ± 0.00
4	100		230	3	8.41 ± 2.61	12.03 ± 3.92	8.04 ± 2.30	1.13 ± 0.67	4.00 ± 0.00	5.00 ± 0.00	3.00 ± 0.00
4	100		230	4	8.41 ± 2.61	12.03 ± 3.92	10.03 ± 3.93	1.45 ± 1.04	4.00 ± 0.00	5.00 ± 0.00	3.00 ± 0.00
4	100		230	5	8.41 ± 2.61	12.03 ± 3.92	11.44 ± 4.07	1.63 ± 1.09	4.00 ± 0.00	5.00 ± 0.00	3.00 ± 0.00
5	100		196	0	7.85 ± 2.07	11.44 ± 3.64	5.94 ± 1.65	0.81 ± 0.34	4.00 ± 0.00	4.77 ± 0.43	3.00 ± 0.00
5	100		196	1	7.85 ± 2.07	10.88 ± 2.90	6.78 ± 1.81	0.92 ± 0.34	4.00 ± 0.00	4.77 ± 0.43	3.00 ± 0.00
5	100		196	2	7.85 ± 2.07	10.88 ± 2.83	7.27 ± 2.11	0.97 ± 0.32	4.00 ± 0.00	4.77 ± 0.43	3.00 ± 0.00
5	100		196	3	7.85 ± 2.07	10.88 ± 2.83	7.99 ± 2.46	1.06 ± 0.34	4.00 ± 0.00	4.77 ± 0.43	3.00 ± 0.00
5	100		196	4	7.85 ± 2.07	10.88 ± 2.83	9.76 ± 2.68	1.32 ± 0.45	4.00 ± 0.00	4.77 ± 0.43	3.00 ± 0.00
5	100		196	5	7.85 ± 2.07	10.88 ± 2.83	11.07 ± 3.67	1.51 ± 0.67	4.00 ± 0.00	4.77 ± 0.43	3.00 ± 0.00
6	100		164	0	4.60 ± 1.18	8.92 ± 3.65	7.81 ± 2.95	1.76 ± 0.66	4.00 ± 0.00	4.93 ± 0.25	2.80 ± 0.61
6	100		164	1	4.60 ± 1.18	6.64 ± 1.92	8.63 ± 3.24	1.95 ± 0.77	4.00 ± 0.00	5.00 ± 0.00	2.80 ± 0.61
6	100		164	2	4.60 ± 1.18	6.53 ± 1.68	11.70 ± 9.62	2.60 ± 2.04	4.00 ± 0.00	5.00 ± 0.00	2.80 ± 0.61
6	100		164	3	4.60 ± 1.18	6.53 ± 1.68	11.05 ± 4.26	2.50 ± 0.92	4.00 ± 0.00	5.00 ± 0.00	2.80 ± 0.61
6	100		164	4	4.60 ± 1.18	6.53 ± 1.68	12.46 ± 5.58	2.78 ± 1.08	4.00 ± 0.00	5.00 ± 0.00	2.80 ± 0.61
6	100		164	5	4.60 ± 1.18	6.53 ± 1.68	15.24 ± 7.24	3.43 ± 1.53	4.00 ± 0.00	5.00 ± 0.00	2.80 ± 0.61
7	100		131	0	3.39 ± 1.12	6.23 ± 1.98	7.58 ± 2.92	2.45 ± 1.15	3.90 ± 0.31	4.53 ± 0.51	3.00 ± 0.00
7	100		131	1	3.39 ± 1.12	4.67 ± 1.48	7.80 ± 2.76	2.53 ± 1.16	4.00 ± 0.00	4.53 ± 0.51	3.00 ± 0.00
7	100		131	2	3.39 ± 1.12	4.65 ± 1.48	8.68 ± 3.34	2.81 ± 1.36	4.00 ± 0.00	4.73 ± 0.45	3.00 ± 0.00
7	100		131	3	3.39 ± 1.12	4.65 ± 1.48	9.19 ± 4.12	2.95 ± 1.54	4.00 ± 0.00	4.83 ± 0.38	3.00 ± 0.00
7	100		131	4	3.39 ± 1.12	4.65 ± 1.48	9.64 ± 2.41	3.10 ± 1.13	4.00 ± 0.00	4.83 ± 0.38	3.00 ± 0.00
7	100		131	5	3.39 ± 1.12	4.65 ± 1.48	12.56 ± 3.82	4.06 ± 1.69	4.00 ± 0.00	4.83 ± 0.38	3.00 ± 0.00
8	100		98	0	2.53 ± 0.50	3.87 ± 0.88	6.45 ± 1.60	2.62 ± 0.77	3.73 ± 0.45	4.83 ± 0.38	2.93 ± 0.25
8	100		98	1	2.53 ± 0.50	3.45 ± 0.65	5.97 ± 1.93	2.44 ± 0.92	3.73 ± 0.45	4.63 ± 0.49	2.80 ± 0.55
8	100		98	2	2.53 ± 0.50	3.46 ± 0.67	6.97 ± 2.08	2.86 ± 1.06	3.80 ± 0.41	4.63 ± 0.49	2.80 ± 0.55
8	100		98	3	2.53 ± 0.50	3.46 ± 0.67	7.91 ± 3.31	3.22 ± 1.42	3.87 ± 0.35	4.70 ± 0.47	2.80 ± 0.55
8	100		98	4	2.53 ± 0.50	3.46 ± 0.67	8.52 ± 2.82	3.49 ± 1.35	3.87 ± 0.35	4.70 ± 0.47	2.80 ± 0.55
8	100		98	5	2.53 ± 0.50	3.46 ± 0.67	9.74 ± 3.10	4.00 ± 1.50	3.87 ± 0.35	4.77 ± 0.43	2.80 ± 0.55
9	80		207	0	3.21 ± 0.53	3.43 ± 0.83	7.09 ± 2.23	2.24 ± 0.78	3.00 ± 0.00	4.00 ± 0.00	2.00 ± 0.00
9	80		207	1	3.21 ± 0.53	3.80 ± 0.65	6.90 ± 1.91	2.20 ± 0.71	3.00 ± 0.00	4.00 ± 0.00	2.00 ± 0.00
9	80		207	2	3.21 ± 0.53	4.53 ± 0.96	8.44 ± 3.05	2.73 ± 1.22	3.00 ± 0.00	4.00 ± 0.00	2.00 ± 0.00
9	80		207	3	3.21 ± 0.53	4.44 ± 0.81	8.58 ± 2.44	2.74 ± 0.93	3.00 ± 0.00	4.00 ± 0.00	2.00 ± 0.00
9	80		207	4	3.21 ± 0.53	4.44 ± 0.81	9.90 ± 1.77	3.46 ± 1.84	3.00 ± 0.00	4.00 ± 0.00	2.00 ± 0.00
9	80		207	5	3.21 ± 0.53	4.44 ± 0.81	10.61 ± 4.72	3.15 ± 0.74	3.00 ± 0.00	4.00 ± 0.00	2.00 ± 0.00
Total		*F*			81.056	58.743	240.444	174.619	145.401	47.455	218.833
		*P*			0.000	0.000	0.000	0.000	0.000	0.000	0.000
A		*F*			102.223	53.688	388.425	281.715	234.941	76.237	349.976
		*P*			0.000	0.000	0.000	0.000	0.000	0.000	0.000
B		*F*			43.142	65.883	0.000	6.484	0.785	1.940	0.118
		*P*			0.000	0.000	1.000	0.000	0.560	0.085	0.988

**Table 7:** CT image quality of small intestine and radiation dose — the summary of portal venous phase

A	Tube voltage (kV)	Tube current (mAs)		B	Objective scoring				Subjective scoring		
					CTDI_vol_ (mGy)	SSDE(mGy)	CNR	CCR	Blood vessel	Intestinal wall	Lesions
1	120	200		0	13.46 ± 0.00	4.60 ± 0.82	4.00 ± 2.00	0.30 ± 0.15	5.00 ± 0.00	5.00 ± 0.00	5.00 ± 0.00
2	100	295		0	7.19 ± 1.24	15.22 ± 4.48	6.87 ± 3.17	1.01 ± 0.59	3.77 ± 0.43	4.77 ± 0.43	3.00 ± 0.00
2	100	295		1	7.19 ± 1.24	9.98 ± 1.95	7.22 ± 3.11	1.07 ± 0.60	3.77 ± 0.43	4.87 ± 0.35	3.00 ± 0.00
2	100	295		2	7.19 ± 1.24	9.98 ± 1.99	7.83 ± 3.44	1.15 ± 0.60	3.77 ± 0.43	5.00 ± 0.00	3.00 ± 0.00
2	100	295		3	7.19 ± 1.24	9.98 ± 1.99	8.73 ± 3.01	1.27 ± 0.51	3.77 ± 0.43	5.00 ± 0.00	3.00 ± 0.00
2	100	295		4	7.19 ± 1.24	9.98 ± 1.99	10.76 ± 5.24	1.60 ± 0.97	3.77 ± 0.43	5.00 ± 0.00	3.00 ± 0.00
2	100	295		5	7.19 ± 1.24	9.98 ± 1.99	10.44 ± 3.95	1.52 ± 0.67	3.77 ± 0.43	5.00 ± 0.00	3.00 ± 0.00
3	100	262		0	7.38 ± 1.56	10.17 ± 2.08	5.95 ± 2.08	0.83 ± 0.30	4.00 ± 0.00	4.57 ± 0.50	3.00 ± 0.00
3	100	262		1	7.38 ± 1.56	10.29 ± 2.48	5.71 ± 2.31	0.80 ± 0.36	4.00 ± 0.00	4.57 ± 0.50	3.00 ± 0.00
3	100	262		2	7.38 ± 1.56	10.34 ± 2.47	6.68 ± 2.46	0.94 ± 0.41	4.00 ± 0.00	4.70 ± 0.47	3.00 ± 0.00
3	100	262		3	7.38 ± 1.56	10.34 ± 2.47	8.03 ± 3.84	1.11 ± 0.52	4.00 ± 0.00	4.70 ± 0.47	3.00 ± 0.00
3	100	262		4	7.38 ± 1.56	10.34 ± 2.47	9.81 ± 5.16	1.34 ± 0.64	4.00 ± 0.00	4.70 ± 0.47	3.00 ± 0.00
3	100	262		5	7.38 ± 1.56	10.34 ± 2.47	10.32 ± 5.76	1.40 ± 0.67	4.00 ± 0.00	4.70 ± 0.47	3.00 ± 0.00
4	100	230		0	6.20 ± 1.16	9.60 ± 2.41	5.30 ± 1.61	0.90 ± 0.36	4.00 ± 0.00	5.00 ± 0.00	3.00 ± 0.00
4	100	230		1	6.20 ± 1.16	8.91 ± 1.80	5.27 ± 1.78	0.89 ± 0.38	4.00 ± 0.00	5.00 ± 0.00	3.00 ± 0.00
4	100	230		2	6.20 ± 1.16	8.87 ± 1.82	6.72 ± 1.55	1.12 ± 0.35	4.00 ± 0.00	5.00 ± 0.00	3.00 ± 0.00
4	100	230		3	6.20 ± 1.16	8.87 ± 1.82	7.67 ± 2.60	1.30 ± 0.58	4.00 ± 0.00	5.00 ± 0.00	3.00 ± 0.00
4	100	230		4	6.20 ± 1.16	8.87 ± 1.82	9.87 ± 4.13	1.67 ± 0.83	4.00 ± 0.00	5.00 ± 0.00	3.00 ± 0.00
4	100	230		5	6.20 ± 1.16	8.87 ± 1.82	10.03 ± 3.14	1.70 ± 0.70	4.00 ± 0.00	5.00 ± 0.00	3.00 ± 0.00
5	100	196		0	5.36 ± 1.17	8.55 ± 1.69	4.23 ± 0.94	0.83 ± 0.26	4.00 ± 0.00	4.77 ± 0.43	3.00 ± 0.00
5	100	196		1	5.36 ± 1.17	7.43 ± 1.57	5.14 ± 1.17	0.99 ± 0.29	4.00 ± 0.00	4.77 ± 0.43	3.00 ± 0.00
5	100	196		2	5.36 ± 1.17	7.46 ± 1.67	5.26 ± 1.34	1.02 ± 0.32	4.00 ± 0.00	4.77 ± 0.43	3.00 ± 0.00
5	100	196		3	5.36 ± 1.17	7.46 ± 1.67	6.54 ± 1.69	1.28 ± 0.45	4.00 ± 0.00	4.77 ± 0.43	3.00 ± 0.00
5	100	196		4	5.36 ± 1.17	7.46 ± 1.67	7.76 ± 2.51	1.52 ± 0.61	4.00 ± 0.00	4.77 ± 0.43	3.00 ± 0.00
5	100	196		5	5.36 ± 1.17	7.46 ± 1.67	8.02 ± 2.36	1.56 ± 0.59	4.00 ± 0.00	4.77 ± 0.43	3.00 ± 0.00
6	100	164		0	4.03 ± 0.78	6.39 ± 1.69	5.82 ± 2.69	1.52 ± 0.82	4.00 ± 0.00	5.00 ± 0.00	2.80 ± 0.61
6	100	164		1	4.03 ± 0.78	5.76 ± 1.02	6.42 ± 2.99	1.65 ± 0.77	4.00 ± 0.00	5.00 ± 0.00	2.80 ± 0.61
6	100	164		2	4.03 ± 0.78	5.70 ± 1.06	7.08 ± 2.97	1.82 ± 0.81	4.00 ± 0.00	5.00 ± 0.00	2.80 ± 0.61
6	100	164		3	4.03 ± 0.78	5.70 ± 1.06	8.13 ± 3.94	2.10 ± 1.08	4.00 ± 0.00	5.00 ± 0.00	2.80 ± 0.61
6	100	164		4	4.03 ± 0.78	5.70 ± 1.06	9.55 ± 3.98	2.46 ± 1.14	4.00 ± 0.00	5.00 ± 0.00	2.80 ± 0.61
6	100	164		5	4.03 ± 0.78	5.70 ± 1.06	9.00 ± 2.97	2.29 ± 0.80	4.00 ± 0.00	5.00 ± 0.00	2.80 ± 0.61
7	100	131		0	3.21 ± 0.69	5.32 ± 1.25	5.60 ± 1.60	1.80 ± 0.58	3.90 ± 0.31	4.53 ± 0.51	3.00 ± 0.00
7	100	131		1	3.21 ± 0.69	4.40 ± 0.84	6.10 ± 2.43	1.94 ± 0.67	4.00 ± 0.00	4.83 ± 0.38	3.00 ± 0.00
7	100	131		2	3.21 ± 0.69	4.40 ± 0.87	6.39 ± 1.55	2.07 ± 0.59	4.00 ± 0.00	4.83 ± 0.38	3.00 ± 0.00
7	100	131		3	3.21 ± 0.69	4.40 ± 0.87	7.13 ± 2.09	2.28 ± 0.69	4.00 ± 0.00	4.83 ± 0.38	3.00 ± 0.00
7	100	131		4	3.21 ± 0.69	4.40 ± 0.87	8.23 ± 2.83	2.64 ± 0.93	4.00 ± 0.00	4.83 ± 0.38	3.00 ± 0.00
7	100	131		5	3.21 ± 0.69	4.40 ± 0.87	9.17 ± 2.92	2.95 ± 0.98	4.00 ± 0.00	4.83 ± 0.38	3.00 ± 0.00
8	100	98		0	2.54 ± 0.49	3.87 ± 0.88	5.02 ± 1.28	2.03 ± 0.59	3.73 ± 0.45	4.63 ± 0.49	2.80 ± 0.55
8	100	98		1	2.54 ± 0.49	3.47 ± 0.63	4.74 ± 1.23	1.93 ± 0.63	3.80 ± 0.41	4.77 ± 0.43	2.93 ± 0.25
8	100	98		2	2.54 ± 0.49	3.47 ± 0.66	5.02 ± 1.45	2.02 ± 0.63	3.87 ± 0.35	4.77 ± 0.43	2.93 ± 0.25
8	100	98		3	2.54 ± 0.49	3.47 ± 0.66	5.59 ± 1.87	2.26 ± 0.80	3.87 ± 0.35	4.83 ± 0.38	2.93 ± 0.25
8	100	98		4	2.54 ± 0.49	3.47 ± 0.66	6.83 ± 2.73	2.75 ± 1.14	3.93 ± 0.25	4.90 ± 0.31	2.93 ± 0.25
8	100	98		5	2.54 ± 0.49	3.47 ± 0.66	7.32 ± 2.37	2.96 ± 1.04	3.87 ± 0.35	4.90 ± 0.31	2.93 ± 0.25
9	80	207		0	3.21 ± 0.54	3.44 ± 0.81	5.73 ± 1.32	1.83 ± 0.52	3.00 ± 0.00	4.00 ± 0.00	2.00 ± 0.00
9	80	207		1	3.21 ± 0.54	3.79 ± 0.65	6.83 ± 2.62	2.21 ± 1.05	3.00 ± 0.00	4.00 ± 0.00	2.00 ± 0.00
9	80	207		2	3.21 ± 0.54	4.52 ± 0.97	6.96 ± 2.15	2.24 ± 0.86	3.00 ± 0.00	4.00 ± 0.00	2.00 ± 0.00
9	80	207		3	3.21 ± 0.54	4.44 ± 0.83	6.04 ± 1.30	1.92 ± 0.50	3.00 ± 0.00	4.00 ± 0.00	2.00 ± 0.00
9	80	207		4	3.21 ± 0.54	4.44 ± 0.83	8.31 ± 1.53	2.65 ± 0.53	3.00 ± 0.00	4.00 ± 0.00	2.00 ± 0.00
9	80	207		5	3.21 ± 0.54	4.44 ± 0.83	8.39 ± 1.54	2.67 ± 0.61	3.00 ± 0.00	4.00 ± 0.00	2.00 ± 0.00
Total			*F*		83.080	37.013	394.085	230.601	162.711	52.341	662.888
			*P*		0.000	0.000	0.000	0.000	0.000	0.000	0.000
A			*F*		97.591	17.037	632.019	368.632	262.511	82.644	1067.243
			*P*		0.000	0.000	0.000	0.000	0.000	0.000	0.000
B			*F*		53.363	65.614	0.000	15.106	0.460	5.120	0.346
			*P*		0.000	0.000	1.000	0.000	0.806	0.007	0.885

#### 3.B.1 Objective assessment of statistical differences in CCR, CNR, CTDIvol, and SSDE between different groups based on group A and B

Statistical difference of the objective assessment was found among groups at each stage (Tables 4, 5, 6, 7). In group A, the CCR of A8 was significantly higher than the other groups (*P *=* *0.000–0.047) according to the result of plain CT scan. The CCR of A6, A7, A8, and A9 was significantly higher than the other groups at the arterial period (*P *=* *0.000–0.001). However, there was no obvious difference among A6, A7, A8 and A9 (*P *=* *0.083–0.676). At the stage of the small intestine period and portal venous phase, the CCR of A7, A8, and A9 was significantly higher than that in other groups (*P *=* *0.000–0.013, *P *=* *0.000–0.003), while there was no obvious difference among the groups of A7–A9 (*P *=* *0.074–0.253, *P *=* *0.452–0.774). With respect to CNR and that of A7, A8, and A9 were obviously higher than the other groups via plain scan (*P *=* *0.000). At the arterial period, the CNR of A1, A2, A5, and A6 was all higher than that of the other groups (*P *=* *0.000–0.049). The CNR of A2 was the highest both at small intestine period and portal venous phase (*P *=* *0.000; *P *=* *0.000–0.003). However, the CNR of A4, A5, A7, A8, and A9 was higher at the small intestine period, while that of A2–A9 at the portal venous phase was lower than that of A1 (*P *=* *0.177–0.826; *P *=* *0.000–0.015).

**Figure 4:**
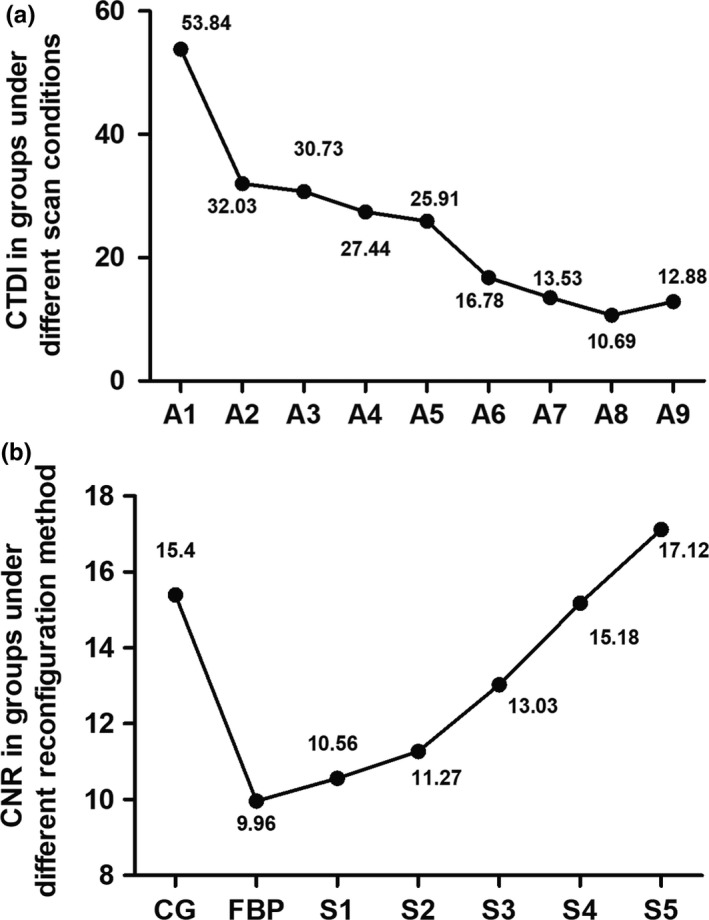
The changes of CTDI and CNR in groups under different scan conditions (a) and reconfiguration method (b) A1‐control group (CG), 120 Kv, 200 mAs, FBP; A2–100 kV, 295 mAs; A3–100 kV, 262 mAs; A4–100 kV, 230 mAs; A5–100 kV, 196 mAs; A6–100 kV, 164 mAs; A7–100 kV, 131 mAs; A8–100 kV, 98 mAs; A9–80 kV, 275 mAs. CG‐control group; FBP: B0; S1: SAFIRE, B1; S2: SAFIRE, B2; S3: SAFIRE, B3; S4: SAFIRE, B4; S5: SAFIRE, B5.

The CTDI_vol_ and SSDE of A7–A9 were all lower than those in other groups at the period of plain CT scan and arterial (*P *=* *0.000). There was no significant difference among three groups except in the plain CT scan (*P *=* *0.297–0.461). At the small intestine period and the portal venous phase, the CTDI_vol_ of A8 was both lower than the other groups (*P *=* *0.000–0.005, *P *=* *0.000) [Fig. 4(a)]. The SSDE of A7, A9, and A1 was closely and lower than the other groups (*P *=* *0.000–0.021) at portal venous phase.

In group B, both of CCR and CNR raised from B0 to B5 in turn. Those of B5 was higher than the other four groups (0.000 < *P *<* *0.05) [Fig. 4(b)]. As the radiation dose did not exchange with the recombination mode in a single scan, the CTDI_vol_ and SSDE were not analyzed.

#### 3.B.2 Subjective assessment of statistical differences between the groups based on group A and B

As respect to subjective assessment, significant difference was observed among group A, but there was no obvious difference among group B (Tables 4, 5, 6, 7). The vascular score of A1 was higher than the other groups at each stage (*P *=* *0.000). Meanwhile, there was no significant difference among group A3–A7 (*P *>* *0.05), and they were both obviously higher than that of group A8 and A9 (*P *=* *0.000). The subjective scoring of intestinal wall in group A1, A2, A4, A5 and A6 were not markedly different (*P *>* *0.05), and higher than the other groups (*P *<* *0.05) at plain CT scan and arterial period. Group A1, A4 and A6 at small intestine period and group A1, A2, A4 and A6 at portal venous phase were higher than the other groups (*P *<* *0.05) and there was significant difference among these groups (*P *<* *0.05). The presentation of lesions manifested that the score of group A1 was significantly higher than other groups at every stage (*P *=* *0.000). Besides, that of group A2, A4, A5, A6 and A7 were all higher than that of group A3, A8 and A9 (*P *=* *0.000) at plain CT scan while group A2–A7 was higher than group A8 and A9 at the other three stages (*P *=* *0.000). However, there was no significant difference among group A2, A4, A5, A6 (*P *=* *0.351–1.000) and A7 as well as A2–A7 at the other three stages (*P *>* *0.05).

The subjective scoring of blood vessel were all higher in group B1–B5 than that in B0 (*P *<* *0.05), and there no significant difference among group B1–B5 at each stage (*P *>* *0.05). The intestinal wall and lesions scoring of group B1–B5 were both higher than that of B0 at plain CT scan and portal venous phase (*P *<* *0.05). At arterial period, the intestinal wall of B2–B5 was higher than that of B0 and B1 while the lesions scoring of B1–B5 was higher than that of B0 (*P *<* *0.05). However, the intestinal wall of B0–B3 was lower than that of B4 and B5 while the lesions scoring of B1–B5 was lower than that of B0 (*P *<* *0.05).

All above, for the patients with standard weight (18.5 kg/m^2 ^≤ BMI ≤ 23.9 kg/m^2^), the better image quality and lower radiation dose could be obtained under the tube voltage of 100 kV and tube current of 131mAs using CAER Dose 4D and SAFIRE 4 or SAFIRE 5 compared to controls (Table 8).

**Table 8:** The best conditions of small intestine CT radiograph

	Tube voltage (kV)	Tube current (mAs)	SAFIRE	CTDI_vol_ (mSv)	*P*
A7	100	131	S4/S5	13.53	<0.001
Benchmark scanning condition	120	200	0	53.84	

## 4 DISCUSSION

There are many factors that influence the radiation dose of CT including scan parameters, hardware and software. The study of reducing CT radiation dose is started from 1980s and the focus is fixed on optimizing scan parameters. The square of the tube voltage is proportional to the radiation dose of x ray and the decrease of it can influence the image noise and spatial resolution.^28^ SAFIRE could measures can data repeatedly and mark noise in the raw data field to reduce the image noise and radiation dose. In our study, we demonstrated that image quality and radiation dose decreased along with the reduction of tube voltage and current when scanning water phantom which was expected. However, as the increase of iterative intensity, the image quality increased. These results are consistent with previous studies.^18^, ^19^, ^20^ There were 26 recombination forms of tube voltage, of which the current satisfied the demands of lower image noise and higher SNR and CNR in comparison to the reference standard. It simplified and optimized the experimental procedure in the following studies significantly.

CARE Dose 4D includes Real‐time Z‐axis tube current modulation and Real‐time angular dose modulation. It can optimize the radiation dose as much as possible via regulating the tube current within certain limits. Previous studies have proved that the radiation dose is reduced and the image noise is increased when CARE Dose 4D is used, and the projection angle, the localizer, protocol selection, patient centering, the use of protective devices, and the scanning direction need to be observed.^22^, ^23^ In current study, the ratio of CNR and CTDI_vol_ (CNR) was assessed for the optimization of image quality and radiation dose. The image quality was much better while the CNR was larger. As CNR with low value is unfavorable for the interpretation and diagnosis of the image, CNR and CTDI_vol_ need to be further analyzed. SSDE put forward by AAPM was used to evaluate the radiation dose of CT. Nine (A) and six groups (B) were divided according to different standard. When the plain CT scan was conducted, the CCR of A8 was the highest, the CNR of A7 and A8 was higher than other groups, while the CTDI_vol_ and SSDE were relatively low. The CCR and CNR of B5 were higher as compared to those in other groups. For the subjective scoring, the display of blood vessel, intestinal wall and lesion of A8 were obviously lower than those of A7. Hence, A7 was best for plain CT scan and combined with SAFIRE 5 for reconstructed images. At the arterial phase, the CCR of A6, A7, and A8 was the highest, the CNR of A6 was higher than that of A7, while the CTDI_vol_ and SSDE of A7 were lower than those in A6. The CCR and CNR of B4 and B5 were higher than those of other groups. The scoring of blood vessel, intestinal wall, and the lesion of A8 were lower than those of A6 and A7 which was little lower than the standard dose. Therefore, A6 was considered as the best choice which could help obtain good image quality and A7 received the lowest radiation dose. Above all, A7 combined with SAFIRE 4 or SAFIRE 5 might be the best choice at this stage. When it was at the small intestine period, the CCR of A7 was highest, and the CNR of A6 was higher than that of A7 and A8, as well as the CTDI_vol_ and SSDE of A8 were lower than other groups. The CCR and CNR of B5 were highest among the group B. The scoring of intestinal wall and the lesion of A8 were all lower than A6 and A7, while that of the blood vessel was slightly lower than that of A6. Therefore, A7 combined with SAFIRE 4 or SAFIRE 5 was recommended as the method to obtain good image quality of small intestine. The CCR of A7, A8, and A9 was all higher than the other groups at portal venous phase, the CNR of A2, A3, A6, and A7 was successively decreased. The CTDI_vol_ and SSDE of A8 were the lowest. The CCR and CNR of B4 and B5 were higher than those in the other groups. The subjective scoring using SARIRE were all higher than using FBP. As a result, the combination of A7 and SAFIRE 4 or SAFIRE 5 was the best condition for the scan. The result was consistent with the study of Andrew et al.^18^ However, the study of Andrew et al. has not compared the interaction of the scan parameter and iterative intensity. Our study used lower scan conditions and higher iterative intensity to ensure the image quality. Besides, the CTDI_vol_ had decreased by 74.85%. Nevertheless, this study still has some limitations. The subjective scoring replaced the objective measurement which might bring some deviation.

## 5 CONCLUSION

In conclusion, the tube voltage of 100 kV, tube current of 131 mAs, and CARE Dose 4D were appropriate for people with a standard weight using a regular CT scan, arterial phase, small intestine period, and a portal venous phase when the MSCT was conducted. The SAFIRE 4 or SAFIRE 5 could achieve images that were of good quality and low dose. The influence of different imaging conditions and postprocessing techniques on the diagnosis accuracy of lesions are still unclear. With the rise in the variety of CT, the combination of them with CARE Dose 4D and iterative recombination technique needs to be further explored.

## CONFLICT OF INTEREST

The authors declare that they have no conflict of interest.
